# Daily tea drinking is not associated with newly diagnosed non-alcoholic fatty liver disease in Chinese adults: the Tianjin chronic low-grade systemic inflammation and health cohort study

**DOI:** 10.1186/s12937-019-0502-y

**Published:** 2019-11-11

**Authors:** Yang Xia, Xuena Wang, Shunmin Zhang, Qing Zhang, Li Liu, Ge Meng, Hongmei Wu, Xue Bao, Yeqing Gu, Shaomei Sun, Xing Wang, Ming Zhou, Qiyu Jia, Kun Song, Qijun Wu, Kaijun Niu, Yuhong Zhao

**Affiliations:** 1 Department of Clinical Epidemiology, Shengjing Hospital of China Medical University, No. 36, San Hao Street, Shenyang, 110004 Liaoning China; 2 Nutritional Epidemiology Institute and School of Public Health, Tianjin Medical University, 22 Qixiangtai Road, Heping District, Tianjin, 300070 Tianjin China; 3 Health Management Centre, Tianjin Medical University General Hospital, Tianjin, China

**Keywords:** Tea consumption, Non-alcoholic fatty liver disease, Prevalence

## Abstract

**Background:**

Previous studies have reported that tea extract supplementation has potential benefits on the risk factors of non-alcoholic fatty liver disease (NAFLD); however, no study has investigated the direct effect of daily tea consumption on the prevalence of NAFLD in the general population. This cross-sectional study aimed to evaluate the associations between tea consumption and the prevalence of newly diagnosed NAFLD among Chinese adults.

**Methods:**

The present cross-sectional study was based on the Tianjin Chronic Low-grade Systemic Inflammation and Health Cohort Study. In total, 19,350 participants were enrolled in the analyses. Tea consumption was assessed via a self-administered food frequency questionnaire. NAFLD was diagnosed via liver ultrasonography and no history of heavy alcohol intake**.** Logistic regression analysis was used to estimate the associations between tea consumption and the prevalence of NAFLD.

**Results:**

Consumption of green tea, oolong tea, and black tea were positively associated with the prevalence of newly diagnosed NAFLD before adjustments. Compared with the participants who never drink tea, the odds ratios (95% confidence interval) of newly diagnosed NAFLD in the highest categories (≥ 1 cup/day) of green tea, oolong tea, black tea, and jasmine tea were 1.48 (1.33, 1.65), 1.50 (1.33, 1.68), and 1.28 (1.13, 1.46), and 1.36 (1.20, 1.54) before adjustments, respectively. However, no significant association was found between tea consumption and the prevalence of NAFLD after adjusting for socio-demographic, behavioural, anthropometric, dietary, and clinical confounding factors.

**Conclusion:**

There is no significant association between daily tea drinking and newly-diagnosed NAFLD in general Chinese adults.

## Introduction

Non-alcoholic fatty liver disease (NAFLD) is rapidly becoming the most common chronic liver disease and potentially affects 25% of the global adult population [1]. NAFLD develops without alcohol abuse and represents a spectrum of liver disease encompassing simple fatty infiltration, fat and inflammation, and cirrhosis [2]. Moreover, NAFLD is associated with metabolic syndrome [3], type 2 diabetes mellitus [4], and chronic kidney disease [5]. However, there is a lack of current evidence regarding the efficacy and safety profiles of pharmacotherapies aimed at treating NAFLD. Lifestyle management, including sustained weight loss, healthy diet, and increased physical activity (PA), is still an important approach to treating NAFLD [6, 7]. Thus, it is important to identify modifiable risk factors and develop preventive strategies to decrease the disease burden of NAFLD.

Tea (*Camellia sinensis* Theaceae) was discovered in China in 3000 BC or earlier [8]. Tea in its various processed forms (i.e., non-fermented green tea, partly-fermented oolong tea, and fermented black tea) represents the most widely consumed beverage worldwide [9]. In Asia, tea has been regarded to process significant health-promoting effects for centuries [10]. Most recently, a meta-analysis that included four randomized controlled trials suggested that there are potential benefits of green tea supplementation on the risk factors of NAFLD [11]. All the studies pooled in that meta-analysis explored the effect of tea consumption on the risk factors of NAFLD by using green tea extract supplementation as treatment [11]. However, daily tea consumption, different from tea extract supplementation, contains lower amounts of tea polyphenol. To the best of our knowledge, no study has explored the associations between daily tea consumption and the prevalence of NAFLD. Thus, we designed this cross-sectional study of the general population to explore the associations between daily tea consumption and the prevalence of newly diagnosed NAFLD among Chinese adults.

## Materials and methods

### Participants

This cross-sectional study was based on the Tianjin Chronic Low-grade Systemic Inflammation and Health (TCLSIHealth) Cohort Study, which is a large prospective dynamic cohort study that focuses on the relationships between chronic low-grade systemic inflammation and the health status of a population living in Tianjin, China [12]. Participants were recruited during their annual health examinations at the Tianjin Medical University General Hospital-Health Management Center and community management centres in Tianjin. This dynamic cohort study was launched in 2007. Furthermore, a detailed lifestyle questionnaire had been administered to about 70–80% of randomly selected subjects from this population since May 2013. The questionnaire covered questions about family income, marital status, employment status, educational level, physical activity, sleep habits, dietary habits, overall computer/mobile device usage time, television time, history of prior infections, and use of medicines as well as physical performance tests.

A total of 32,165 participants without acute inflammatory disease completed a comprehensive health examination and the study questionnaire. We excluded participants who changed their lifestyles (including dietary intake, drinking, smoking, physical activity, and sleeping habits) (assessed via a question in the questionnaire) in the last five years (*n* = 7364), or those had a history of cardiovascular disease (*n* = 1057), cancer (*n* = 173), NAFLD (*n* = 3344), alcoholic fatty liver disease (*n* = 775), and other live diseases (including chronic hepatitis B or C, autoimmune liver disease, and liver surgery, *n* = 102). The final study population comprised 19,350 individuals.

### Assessment of tea consumption

Dietary intake was assessed using a validated food frequency questionnaire (FFQ) that included 100 food items with specified serving sizes. The FFQ included seven frequency categories ranging from ‘almost never eat’ to ‘twice or more per day’ for foods and eight frequency categories ranging from ‘almost never drink’ to ‘four or more times per day’ for beverages. The reproducibility and validity of the questionnaire have been assessed in a random sample of 150 participants living in Tianjin by comparing the data from the questionnaire with the data from two dietary questionnaires collected approximately three months apart and four-day weighed dietary records (WDRs). Spearman’s rank correlation coefficient for energy intake between two food frequency questionnaires administered three months apart was 0.68. The correlation coefficients for food items (fruits, vegetables, fish, meat, and beverages) between two food frequency questionnaires administered three months apart ranged from 0.62 to 0.79. Spearman’s rank correlation coefficient for energy intake as assessed via the WDRs and FFQ was 0.49. Correlation coefficients for nutrients (vitamin C, vitamin E, polyunsaturated fats, saturated fats, carbohydrates and calcium) as assessed via the WDRs and FFQ ranged from 0.35 to 0.54.

The mean daily intake of nutrients was calculated using an ad hoc computer program developed to analyse the questionnaire. The Chinese food composition tables [13] were used as the nutrient database. Factor analysis was applied to generate the major dietary patterns and food loading on all 100 food items and beverages (gram). Factors were named descriptively according to the food items that showed high loading (absolute value > 0.3) with respect to each dietary pattern as follows: sweet foods pattern, vegetable pattern and animal foods pattern (Additional file 1: Table S1). The scores of dietary patterns were used for further analyses as confounding factors.

The participants indicated the frequency of consumption of green tea, oolong tea, black tea, and jasmine tea over the previous one month in terms of the specified serving size by selecting one of the following eight frequency categories: almost never, < 1 cup/week, 1 cup/week, 2–3 cups/week, 4–6 cups/week, 1 cup/day, 2–3 cups/day, and ≥ 4 cups/day (200 ml per cup). In the study region, a cup of tea has a typical volume of 200 ml. In the present study, the frequency of intake of green tea, oolong tea, and black tea were categorised as: almost never, < 1 cup/week, 1–6 cups/week and ≥ 1 cup/day.

### Liver ultrasonography and definitions of NAFLD

Liver ultrasonography was performed by trained sonographers using a TOSHIBA SSA-660A ultrasound machine (Toshiba, Tokyo, Japan) with a 2–5–MHz curved array probe. According to the revised definition and treatment guidelines for NAFLD published by the Chinese Association for the Study of Liver Disease in 2010 [14], we defined ‘heavy drinking’ as an intake of > 140 g of alcohol per week among men and > 70 g per week among women. Total alcohol intake in the preceding week was assessed using the FFQ. Participants were diagnosed as having NAFLD if they had evidence on abdominal ultrasonography (brightness of liver and a diffusely echogenic change in the liver parenchyma) and no history of heavy drinking.

### Assessment of other variables

The educational level was assessed by asking the question ‘what is the highest degree you earned?’ with two possible response categories: <college graduate or ≥ college graduate. Employment status was classified as either senior officials and managers or professionals. Information on smoking (‘never,’ ‘former,’ and ‘current smoking’) and drinking (‘never,’ ‘former,’ ‘current drinking everyday’, and ‘current drinking sometimes’) status of the participants was obtained via a questionnaire survey. PA in the most recent week was assessed using the short form of the International Physical Activity Questionnaire [15]. The questionnaire asked whether subjects had performed any activities from the following categories during the preceding week: walking, moderate activity (household activity or child care), and vigorous activity (running, swimming, or other sporting activities). Metabolic equivalents (METs; hours per week) were calculated using corresponding MET coefficients (3.3, 4.0, and 8.0, respectively) according to the following formula: MET coefficient of activity × duration (hours) × frequency (days). Total PA levels were assessed by combining separate scores for different activities. Weight and height were assessed using an automatic height and weight measurement instrument (Omron HNH-219; Omron, Kyoto, Japan) with a standard protocol. Body mass index (BMI) was calculated by dividing the weight in kilograms by the square of the height in meters (kg/m^2^). Fasting blood samples were obtained via venipuncture of the cubital vein and immediately mixed with EDTA. Waist circumference was measured at the umbilical level with participants standing and breathing normally. Blood pressure was measured twice from the upper left arm using a TM-2655P automatic device (A&D Co., Tokyo, Japan) after five minutes of rest in a seated position. The mean of these two measurements was taken as the blood pressure value. Hypertension was defined as average systolic BP ≥ 140 mmHg or average diastolic BP ≥90 mmHg or use of antihypertension medications [16]. Blood samples for the analysis of fasting blood glucose and lipid levels were collected in siliconised vacuum plastic tubes. Fasting blood sugar level was measured via the glucose oxidase method, triglyceride level was measured via enzymatic methods, and high-density lipoprotein cholesterol level was measured via the chemical precipitation method using reagents obtained from Roche Diagnostics on an automatic biochemistry analyser (Roche Cobas 8000 modular analyser; Roche, Mannheim, Germany). HbA1c separation and quantification were conducted by a high-performance liquid chromatography analyzer (HLC-723 G8; Tosoh, Tokyo, Japan). In addition, an oral glucose tolerance test was performed, and postprandial glucose was determined in blood samples obtained 2 h after oral administration of a standard 75 g glucose solution. Type 2 diabetes was defined as having fasting blood glucose ≥7.0 mmol/, or 2-h postprandial blood glucose ≥11.1 mmol/l, or HbA1c ≥ 6.5% (48 mmol/mol), or a history of diabetes based on the 2014 American Diabetes Association criteria [17]. Hyperlipidemia was defined as TC ≥ 5.20 mmol/L, or TG ≥ 1.70 mmol/L, or self-reported clinically diagnosed hyperlipidemia according to 2016 Chinese guidelines for the management of dyslipidemia in adults [18].

### Statistical analysis

In the assessment of characteristics of participants according to NAFLD status, continuous variables have been presented as the least square mean (with 95% confidence interval, [CI]) and examined using analysis of variance. Categorical variables have been presented as percentages and examined using chi-square test. Associations between tea drinking and the prevalence of newly diagnosed NAFLD were assessed via conditional logistic regression analysis. Odds ratios (ORs) and 95% CIs were calculated. All *P* values for linear trend were calculated according to the categories of tea consumption (almost never: 1, < 1 cup/week: 2, 1–6 cups/week: 3, ≥1 cup/day: 4) based on logistic regression. Model 1 was adjusted for age, BMI, and sex. Model 2 was additionally adjusted for energy intake (kJ/d), type 2 diabetes, hypertension, hyperlipidemia, physical activity, educational level, household income, smoking status, drinking status, employment status, family history of cardiovascular disease, cancer, and diabetes, intake of sweet foods pattern, vegetable pattern and animal foods pattern, and consumption of two other kinds of tea. All analyses were performed using the Statistical Analysis System 9.3 edition for Windows (SAS Institute Inc., Cary, NC, USA) and STATA (version 12.1; Stata Corp LP, College Station, TX, USA). All *P*-values were two-tailed and differences with *P* < 0.05 were considered statistically significant.

## Results

### Characteristics of participants

Table 1 shows the age- and sex-adjusted characteristics according to NAFLD status. Among the 19,350 participants, 19.4% were classified as newly diagnosed NAFLD. After adjustments for age and sex where appropriate, participants with NAFLD tended to be men, older, of lower education level, less likely to be employed as managers (*P* < 0.0001), and current or ex-smokers, who drank sometimes, had lower daily energy intake and lower intake of vegetable pattern (*P* < 0.0001), had a higher prevalence of type 2 diabetes, hypertension, hyperlipidaemia, and family history of diabetes, and higher BMI and alanine aminotransferase level (*P* < 0.0001).

**Table 1:** Age- and sex- adjusted characteristics according to NAFLD status ^a^

Characteristics	NAFLD status		*P* value ^b^
	No (*n* = 15,589)	Yes (*n* = 3761)	
Sex (male %)	46.6	81.6	< 0.0001
Age (y)	37.5 (37.4, 37.7) ^a^	41.3 (41.0, 41.7)	< 0.0001
BMI	22.9 (22.8, 23.0)	27.2 (27.1, 27.3)	< 0.0001
Physical activity (Mets × hours/week)	10.5 (10.2, 10.7)	10.2 (9.8, 10.6)	0.31
Energy intake (kJ/d)	8457.9 (8420.3, 8496.0)	8257.0 (8180.4, 8334.9)	< 0.0001
Education (≥college graduate, %)	71.2	59.9	< 0.0001
Household income (≥10,000 Yuan, %)	33.4	31.6	0.04
ALT (U/L)	15.3 (15.2, 15.4)	25.0 (24.6, 25.4)	< 0.0001
Type 2 diabetes (%)	1.3	8.4	< 0.0001
Hypertension (%)	12.7	40.3	< 0.0001
Hyperlipidaemia (%)	31.1	66.3	< 0.0001
Dietary pattern score (multiply by 100)			
Sweets foods pattern	0.3 (−1.3, 1.9)	−2.4 (− 5.7, 0.9)	0.15
Vegetable pattern	2.2 (0.7, 3.8)	−6.4 (−9.6, − 3.1)	< 0.0001
Animal foods pattern	1.7 (0.2, 3.3)	−2.1 (− 5.3, 1.1)	0.04
Smoking status (%)			
Smoker	14.0	27.6	< 0.0001
Ex-smoker	3.5	6.2	< 0.0001
Non-smoker	82.5	66.2	< 0.0001
Drinker (%)			
Everyday	3.6	2.1	< 0.0001
Sometime	52.6	61.7	< 0.0001
Ex-drinker	10.3	12.4	< 0.001
Non-drinker	33.4	23.7	< 0.0001
Employment status (%)			
Managers	44.6	39.7	< 0.0001
Professionals	16.0	16.7	0.29
Other	39.5	43.6	< 0.0001
Family history of diseases (%)			
CVD	26.0	22.4	< 0.0001
Hypertension	46.6	47.3	0.44
Diabetes	23.1	27.7	< 0.0001

^a^ NAFLD, non-alcoholic fatty liver disease; CVD, cardiovascular disease; BMI, body mass index; ALT, alanine aminotransferase

^b^ Analysis of variance or chi-square test with adjustments for age and sex where is appropriate

^c^ Least square mean (95% confidence interval) (all such values)

### Tea consumption and NAFLD

The associations between tea consumption and the prevalence of newly diagnosed NAFLD are shown in Table 2. Compared with the participants who never drank tea, the ORs (95% CI) of newly diagnosed NAFLD in the highest category (≥1 cup/day) of green tea, oolong tea, black tea, and jasmine tea before adjustments were1.48 (1.33, 1.65), 1.50 (1.33, 1.68), 1.28 (1.13, 1.46), and 1.36 (1.20, 1.54), respectively. However, no significant association was found between tea consumption and NAFLD after adjustment for socio-demographic, behavioural, anthropometric, dietary, and clinical confounding factors.

**Table 2:** Association between tea consumption and NAFLD ^*^

Tea	Categories of tea consumption (*n* = 19,350)				*P* for trend ^a^
	Almost never	< 1 cup/week	1–6 cups/week	≥ 1 cup/day	
Green tea					
No. of participants	6716	4969	4764	2910	
No. of NAFLD patients (%)	1198 (17.84)	881 (17.73)	976 (20.49)	706 (24.34)	
Crude model	Ref	0.99 (0.90, 1.09) ^b^	1.19 (1.08, 1.30)	1.48 (1.33, 1.65)	< 0.0001
Adjusted model 1 ^c^	Ref	0.99 (0.88, 1.12)	0.98 (0.87, 1.11)	0.99 (0.86, 1.13)	0.77
Adjusted model 2 ^d^	Ref	1.05 (0.90, 1.22)	1.04 (0.89, 1.20)	1.04 (0.88, 1.22)	0.65
Oolong tea					
No. of participants	9083	4937	3551	1779	
No. of NAFLD patients (%)	1669 (18.37)	895 (18.13)	749 (21.09)	448 (25.18)	
Crude model	Ref	0.98 (0.90, 1.08)	1.19 (1.08, 1.31)	1.50 (1.33, 1.68)	< 0.0001
Adjusted model 1 ^c^	Ref	0.94 (0.84, 1.05)	0.95 (0.84, 1.07)	1.05 (0.90, 1.22)	0.99
Adjusted model 2 ^d^	Ref	0.92 (0.79, 1.07)	0.97 (0.83, 1.15)	1.04 (0.86, 1.25)	0.80
Black tea					
No. of participants	9227	5154	3350	1619	
No. of NAFLD patients (%)	1740 (18.86)	968 (18.78)	681 (20.33)	372 (22.98)	
Crude model	Ref	1.00 (0.91, 1.09)	1.10 (0.99, 1.21)	1.28 (1.13, 1.46)	< 0.001
Adjusted model 1 ^c^	Ref	1.04 (0.93, 1.16)	0.90 (0.79, 1.02)	1.04 (0.89, 1.22)	0.57
Adjusted model 2 ^d^	Ref	1.10 (0.96, 1.26)	0.91 (0.78, 1.07)	0.99 (0.83, 1.19)	0.42
Jasmine tea					
No. of participants	10,736	4764	2341	1509	
No. of NAFLD patients (%)	2033 (18.94)	849 (17.82)	515 (22.00)	364 (24.12)	
Crude model	Ref	0.93 (0.85, 1.01) ^b^	1.21 (1.08, 1.35)	1.36 (1.20, 1.54)	< 0.001
Adjusted model 1 ^c^	Ref	0.91 (0.82, 1.02)	0.98 (0.85, 1.12)	0.96 (0.82, 1.13)	0.46
Adjusted model 2 ^d^	Ref	0.90 (0.79, 1.03)	1.06 (0.90, 1.24)	0.93 (0.78, 1.11)	0.68

^*^ NAFLD, non-alcoholic fatty liver disease;

^a^ Multiple conditional logistic regression analysis

^b^ Odds ratios (95% confidence interval) (all such values)

^c^ Adjusted for age, BMI, and sex

^d^ Adjusted for age, BMI, sex, energy intake (kJ/d), type 2 diabetes, hypertension, hyperlipidaemia, physical activity, educational level, household income, smoking status, drinking status, employment status, family history of CVD, cancer, and diabetes, intake of sweet foods pattern, vegetable pattern and animal foods pattern, and consumption of other two kinds of tea

## Discussion

The present study, to the best of our knowledge, is the first to explore the associations between daily tea consumption and the prevalence of newly-diagnosed NAFLD in a general population with a large sample size. Sociodemographic, behavioural, metabolic, and health status confounding factors were adjusted in logistic regression to explore the associations between daily tea consumption and the prevalence of NAFLD. Furthermore, we also adjusted scores of major dietary patterns in the study population in case the associations between daily tea consumption and the prevalence of NAFLD may be affected by intake of dietary confounding factors (i.e. high consumption of red meat [19], fruits [20], sweet products, desserts, and sugar [21]). No significant association was detected between daily consumption of green tea, oolong tea, black tea, and jasmine tea and the prevalence of newly diagnosed NAFLD in Chinese adults after adjustments.

Previous studies have focused on the effect of tea extract on NAFLD. A previous meta-analysis of four clinical trials suggested that green tea extract supplementation yield benefits on NAFLD related risk factors [11]. The results indicated green tea extract supplementation significantly altered the blood levels of alanine and aspartate aminotransferases [11]. Meanwhile, green tea extract supplementation was found to yield a favourable effect on BMI, triacylglycerol, total cholesterol, and low-density lipoprotein cholesterol [11]. Previous studies also suggested that green tea extract could improve obesity, hyperlipidaemia, and NAFLD in mice [22]. The mechanisms underlying how green tea protects against NAFLD are mostly related to catechins present in tea. The evidence from in vitro and in vivo studies indicates that green tea inhibits dietary lipid absorption [23], decreases lipid accumulation in the liver and adipose tissue [24], improves insulin sensitivity [25], and exerts antioxidant properties [26].

However, no study has reported on the beneficial effect of daily tea consumption and the prevalence of NAFLD. We found a positive association between consumption of tea and the prevalence of newly-diagnosed NAFLD before adjustments. This may be due to the fact that participants who drank tea tended to be men and older. Meanwhile, men and older have higher prevalence of NAFLD than women and younger participants [27]. There was no association between tea consumption and NAFLD after adjustment of confounding factors. The results are not in line with those of previous studies which found that tea extracts are associated with a lower prevalence of the risk factors of NAFLD. Daily tea consumption yields a lower content of catechins than in tea extract. A previous meta-analysis showed that catechin intake is associated with a lower prevalence of NAFLD risk factors [11], and that the daily intake amounts of catechin in the treatment groups of previous randomised clinical trials were > 500 mg. However, the amounts of catechins in most commercially available green teas is about 60 mg per 200 ml [28], which is equal to one cup in the present study. Thus, the low amount of catechins consumed daily in tea may not be enough to have a beneficial effect on NAFLD. Moreover, a previous study conducted among Chinese women showed that tea consumption is associated with the serum concentration of total organochlorine pesticides (r = 0.14, *P* < 0.05) [29]. A previous study demonstrated that green tea consumption was positively associated with the risk of type 2 diabetes in Chinese adults (hazard ratio: 1.20; 95% CI: 1.14, 1.27) [30]. Liu et al. suggested that pesticide contamination of tea could underlie the association between daily tea consumption and the incidence of type 2 diabetes [30]. Significant increases in serum biochemical indices, including the levels of alanine aminotransferases, aspartate aminotransferase, alkaline phosphatase, total cholesterol, triglycerides, low-density lipoprotein cholesterol, and lipid peroxidation by-products have been observed experimentally after subchronic or chronic exposure to organophosphates [31, 32]. Thus, it is possible that the pesticide residue in tea, released into hot water could attenuate the beneficial effect of tea in the present study. Further studies are warranted to directly assess pesticide levels in tea and confirm the findings of the present study.

It is also possible that the participants with NAFLD have changed their habit of tea consumption to achieve healthy lifestyles. Thus, the reverse causation may partly explain the findings of the present study. However, we excluded participants who had changed their lifestyles (including dietary intake, drinking, smoking, activity, and sleeping) in the preceding five years. Moreover, we also excluded participants who had a history of NAFLD. The participants with NAFLD in the present study are newly diagnosed and have not changed their habits of tea consumption in the preceding five years. Thus, reverse causation was corrected as much as possible. Another strength of the present study is that although a previous study indicated that tea extract supplementation might yield beneficial effects in patients with NAFLD [11], there is limited evidence for the relationship between daily tea consumption and the prevalence of NAFLD in the general population. Although the underlying mechanism remains unclear, the present study extended the evidence in this regard, and found no association between daily tea consumption and the prevalence of newly diagnosed NAFLD among Chinese adults.

The present study has some limitations. First, due to the nature of the self-reporting questionnaire, recall bias existed and the amount of tea consumed may not have been precisely reported. Second, we excluded participants with health conditions; thus, the final sample may not be representative of the population. However, it is reasonable to exclude participants with cardiovascular disease and cancer when estimating the associations between tea consumption and NAFLD. Third, although we considered as many covariates as possible, we cannot rule out the possibility that unmeasured factors might contribute to the associations observed. Fourth, all the previous studies on tea and NAFLD showed effects at dosage higher than 500 mg of catechins/day (equivalent to 9 cups of green tea). It is possible that the lack of association is due to the fact that the proportion of participants who drink enough cups of tea per day is too low in a general population. Further randomized controlled trials are needed to explore the associations between excessive tea drinking and the prevalence of NAFLD. Fifth, the validity of the short form of IPAQ has not been tested in the study population and the physical activity may not be exact. Finally, liver biopsy, the gold standard for the diagnosis of liver disease, was not performed in the present study, due to the apparently healthy study population. Instead, we diagnosed fatty liver via hepatic ultrasonography. This technique has a sensitivity of 89% and a specificity of 93% and is widely used in population-based studies due to its noninvasiveness and easy accessibility [33].

## Conclusions

Despite the limitations, the results of the present study demonstrate that consumption of tea (≥ 1 cup/day) is not associated with the prevalence of newly diagnosed NAFLD among general Chinese adults. Further studies are needed to explore the associations between more cups of tea drinking (e.g., equivalent to 500 mg of catechins/day) and the prevalence of NAFLD.

## Supplementary information

**Additional file 1: Table S1.** The factor loadings scores of primary food groups of dietary patterns.

## Data Availability

The datasets generated and/or analysed during the current study are not publicly available due to that it is an ongoing cohort study but are available from the corresponding author on reasonable request.
