# Supplementary material for: Daily tea drinking is not associated with newly diagnosed non-alcoholic fatty liver disease in Chinese adults: the Tianjin chronic low-grade systemic inflammation and health cohort study
Source: Nutr J. 2019 Nov 11;18:71. doi: 10.1186/s12937-019-0502-y (PMC6849323; doi:10.1186/s12937-019-0502-y)
Supplement: Supplementary file 1 — Additional file 1: Table S1. The factor loadings scores of primary food groups of dietary patterns. [file 12937_2019_502_MOESM1_ESM.docx]

| Table S1. The factor loadings scores **^*^** of primary food groups of dietary patterns | | | | | |
| --- | --- | --- | --- | --- | --- |
| **Sweets foods pattern** | | **Vegetable pattern** | | **Animal foods pattern** | |
| **Food items** | **Factor loadings** | **Food items** | **Factor loadings** | **Food items** | **Factor loadings** |
| Strawberry, kiwi fruit, persimmon | 0.59 | Chinese cabbage | 0.62 | Animal offal (except for animal liver) | 0.62 |
| Sweets, candied fruits | 0.56 | Celery | 0.61 | Animal liver | 0.58 |
| Chinese cakes | 0.56 | Cucumber | 0.60 | Animal blood | 0.58 |
| Pineapple | 0.55 | Green vegetable | 0.58 | Preserved egg | 0.55 |
| Western-style pastry, cakes | 0.54 | Pumpkin, carrot | 0.56 | Sausage | 0.52 |
| Peach | 0.51 | Eggplant | 0.54 | Instant noodle | 0.50 |
| Cookies | 0.50 | Chinese watermelon | 0.52 | Sea fish | 0.48 |
| Grape | 0.50 | Tomato (including the ketchup) | 0.49 | Pork skin | 0.47 |
| Ice cream | 0.49 | Mushroom | 0.48 | Wonton | 0.46 |
| Watermelon | 0.48 | Raw vegetables (except for tomato) | 0.48 | Freshwater fish | 0.45 |
| Salted eggs | 0.48 | Bell peppers | 0.46 | Seafood (shellfish, squid, shrimp) | 0.43 |
| Pear | 0.47 | Potato (except for sweet potato) | 0.46 | Miscellaneous sauce noodles | 0.41 |
| Chinese sauerkraut | 0.45 | Radish (expect for carrot) | 0.46 | Steamed stuffed bun, dumpling | 0.38 |
| Preserved bean curd | 0.45 | Soya bean products | 0.43 | Bread | 0.34 |
| Banana | 0.44 | Sweet potato | 0.41 | Poultry | 0.32 |
| Sea-plant | 0.44 | Coarse cereals | 0.41 | Carbonated beverage | 0.31 |
| Other kinds of fruit | 0.42 | Congee | 0.39 | Low-fat milk | 0.30 |
| Leek | 0.41 | Hot pepper | 0.38 | Chinese watermelon | 0.28 |
| Onion | 0.40 | Egg | 0.38 | Fruit juice, vegetable juice | 0.27 |
| Lotus root | 0.39 | Ginger | 0.37 | Meat | 0.27 |
| **^*^** For simplicity, only the top 20 food groups of factor loading scores of each pattern are shown. | | | | | |
